# Influence of Post-Traumatic Stress Disorder on Neuroinflammation and Cell Proliferation in a Rat Model of Traumatic Brain Injury

**DOI:** 10.1371/journal.pone.0081585

**Published:** 2013-12-09

**Authors:** Sandra A. Acosta, David M. Diamond, Steven Wolfe, Naoki Tajiri, Kazutaka Shinozuka, Hiroto Ishikawa, Diana G. Hernandez, Paul R. Sanberg, Yuji Kaneko, Cesar V. Borlongan

**Affiliations:** 1 Center of Excellence for Aging and Brain Repair, Department of Neurosurgery and Brain Repair, University of South Florida College of Medicine, Tampa, Florida, United States of America; 2 James A. Haley Veterans Affairs Medical Center, Tampa, Florida, United States of America; 3 Department of Psychology, Center for Preclinical & Clinical Research on PTSD, Department of Molecular Pharmacology and Physiology, University of South Florida, Tampa, Florida, United States of America; 4 Office of Research and Innovation, University of South Florida, Tampa, Florida, United States of America; Georgia Health Sciences University, United States of America

## Abstract

Long-term consequences of traumatic brain injury (TBI) are closely associated with the development of severe psychiatric disorders, such as post-traumatic stress disorder (PTSD), yet preclinical studies on pathological changes after combined TBI with PTSD are lacking. In the present in vivo study, we assessed chronic neuroinflammation, neuronal cell loss, cell proliferation and neuronal differentiation in specific brain regions of adult Sprague-Dawley male rats following controlled cortical impact model of moderate TBI with or without exposure to PTSD. Eight weeks post-TBI, stereology-based histological analyses revealed no significant differences between sham and PTSD alone treatment across all brain regions examined, whereas significant exacerbation of OX6-positive activated microglial cells in the striatum, thalamus, and cerebral peduncle, but not cerebellum, in animals that received TBI alone and combined TBI-PTSD compared with PTSD alone and sham treatment. Additional immunohistochemical results revealed a significant loss of CA3 pyramidal neurons in the hippocampus of TBI alone and TBI-PTSD compared to PTSD alone and sham treatment. Further examination of neurogenic niches revealed a significant downregulation of Ki67-positive proliferating cells, but not DCX-positive neuronally migrating cells in the neurogenic subgranular zone and subventricular zone for both TBI alone and TBI-PTSD compared to PTSD alone and sham treatment. Comparisons of levels of neuroinflammation and neurogenesis between TBI alone and TBI+PTSD revealed that PTSD did not exacerbate the neuropathological hallmarks of TBI. These results indicate a progressive deterioration of the TBI brain, which, under the conditions of the present approach, was not intensified by PTSD, at least within our time window and within the examined areas of the brain. Although the PTSD manipulation employed here did not exacerbate the pathological effects of TBI, the observed long-term inflammation and suppressed cell proliferation may evolve into more severe neurodegenerative diseases and psychiatric disorders currently being recognized in traumatized TBI patients.

## Introduction

Approximately 2 million Americans every year suffer traumatic brain injury (TBI) [1]. Due to medical advances, the mortality rate associated with TBI has declined from 24.9 per 100,000 US residents in 1979 to 17.8 per 100,000 US residents in 2007 [2], [3]. However, an estimated 90,000 survivors will experience loss of physical and cognitive functions [4]. As a consequence, there is an increase of TBI-related chronic illnesses such as memory impairments and, neuropsychological disabilities including depression, anxiety, and post-traumatic stress disorder (PTSD), which impedes quality of life and contributes to a high cost of disability annually [4], [5]. These TBI-induced neuropsychological disabilities either persist or develop late in life and may precipitate anxiety disorders and PTSD in veterans and civilians [6], [7], [8], [9]. However, there is no clear evidence on how these psychiatric morbidities interact with chronic TBI [6].

Accumulating evidence indicates TBI closely presents with neurological impairments, which progressively worsen over time, and lead to secondary injuries instigating a diffused neuroinflammatory response [1], [5], [10], [11], [12] and neurogenic alterations [13], [14], [15]. Although these early immunological and neural disturbances are becoming recognized in the laboratory, the long-term pathological consequences of TBI have remained underexplored. In particular, whether traumatic stress at the time of TBI exacerbates chronic neuroinflammation and suppressed neurogenesis is not fully understood. To this end, the present in vivo study recognized the gap in knowledge on the pathological link between TBI and PTSD, and embarked on characterizing the neuroinflammatory response, neuronal cell loss, cell proliferation and neuronal differentiation by integrating an animal model of chronic TBI with a well-established animal model of PTSD [16], [17], [18]. The emergence of PTSD as a major co-morbidity factor associated with TBI is an urgent clinical unmet need. Because TBI has become the signature wound of wars in Iraq and Afghanistan, improving the clinical outcome will most likely require treating TBI, as well as co-morbid disorders, including PTSD.

## Materials and Methods

### Subjects

Experimental procedures were approved by the University of South Florida Institutional Animal Care and Use Committee (IACUC). All animals were housed under ambient conditions (20°C, 50% relative humidity, and a 12-h light/dark cycle), and necessary precautions were undertaken throughout the study to minimize pain and stress associated with the experimental treatments. All studies were performed by personnel blinded to the treatment conditions.

### TBI surgical procedures

Ten-week old Sprague–Dawley rats (*n* = 24) were subjected to either moderate TBI using a controlled cortical impactor (CCI) (n = 12, n = 6 TBI alone and n = 6 TBI-PTSD) or sham treatment (no TBI) (n = 6 sham surgery-no PTSD and n = 6 sham surgery-PTSD). Deep anesthesia was achieved using 1–2% isoflurane, and it was maintained using a gas mask. All animals were fixed in a stereotaxic frame (David Kopf Instruments, Tujunga, CA, USA). After exposing the skull, the CCI rod impacted the brain at the fronto-parietal cortex (coordinates of −0.2 mm anterior and +0.2 mm lateral to the midline) with a velocity of 6.0 m/s reaching a depth of 1.0 mm below the dura and remained in the brain for 150 milliseconds. The CCI rod was angled 15° degrees vertically to maintain a perpendicular position in reference to the tangential plane of the brain curvature at the impact surface. A linear variable displacement transducer (Macrosensors, Pennsauken, NJ), which was connected to the impactor, measured the velocity and duration to verify consistency across animals. Sham control injury surgeries (i.e., uninjured animals) consisted of animals exposed to anesthesia, scalp incision, craniectomy, and suturing. An electric drill was used to performed the craniectomy of about 4 mm radius centered from bregma −0.2 anterior and +0.2 mm lateral right. A computer operated thermal blanket pad and a rectal thermometer allowed maintenance of body temperature within normal limits. All animals were closely monitored post-operatively with weight and health surveillance recording as per IACUC guidelines. Rats were kept hydrated at all times, and the analgesic ketoprofen was administered after TBI surgery and as needed thereafter. Pre and post TBI, rats were fed with regular rodent diet from Harlan (Harlan 2018).

### Post-traumatic stress disorder regimen

Rats were exposed to an adult cat for 1 hr on two occasions, separated by 10 days (Days 1 and 11). They experienced non-tactile (visual, olfactory, auditory) cues of the cat in our model of PTSD, as we described previously [16], [17], [18], [19], [20], [21]. Social instability was produced with pseudorandom changes in the pairs of cage cohorts on a daily basis. Rats experienced social instability for a total of 31 days (Days 1–31). Therefore, the two cat exposures overlapped with the period of social instability. The combination of social instability and cat exposure produces remarkable PTSD-like behavioral, physiological, endocrine and pharmacological abnormalities in stressed rats [16], [17], [18]. The TBI surgical procedure occurred one day following the second cat exposure. Thus, TBI was induced 11 days after stress induction, and then post-TBI recovery took place during a subsequent period of 20 days of stress. This approach was designed to mimic battlefield conditions in which TBI occurs in already stressed soldiers, and then their recovery must occur in conjunction with post-TBI stress.

### Hematoxylin and eosin analysis

Under deep anesthesia, rats were euthanized at 8 weeks after TBI surgery, and perfused through the ascending aorta with 200 ml of ice cold phosphate buffer saline (PBS), followed by 200 ml of 4% paraformaldehyde (PFA) in PBS. H&E staining was performed to confirm the core impact injury of our TBI model. As shown in our previous studies [22], [23], [24], we demonstrated primary damage to the fronto-parietal cortex. Lesion for impacted area is approximately 29.6±9.7 mm^2^. In addition, H&E staining was analyzed in the hippocampus. Starting at coordinates AP-2.0 mm and ending AP-3.8 mm from bregma, coronal brain sections (40 µm) covering the dorsal hippocampus were selected. A total of 6 sections per rat was used (n = 3 randomly selected rats per group). Cells presenting with nuclear and cytoplasmic staining (H&E) were manually counted in the CA3 neurons. CA3 cell counting spanned the whole CA3 area, starting from the end of hilar neurons to the beginning of curvature of the CA2 region in both the ipsilateral and contralateral side. Sections were examined with Nikon Eclipse 600 microscope at 20X.

### Immunohistochemistry

Under deep anesthesia, rats were sacrificed 8 weeks after TBI surgery, and perfused through the ascending aorta with 200 ml of ice cold phosphate buffer saline (PBS), followed by 200 ml of 4% paraformaldehyde (PFA) in PBS. Brains were removed and post-fixed in the same fixative for 24 hours followed by 30% sucrose in phosphate buffer (PB) for 1 week. Coronal sectioning was carried out at a thickness of 40 µm by cryostat. Staining for the cell cycle–regulating protein Ki67, migrating neuronal marker DCX, and activated microglial cell markers OX6 was done on every sixth coronal section throughout the entire striatum and dorsal hippocampus. Sixteen free-floating coronal sections (40 µm) were incubated in 0.3% hydrogen peroxide (H_2_O_2_) solution followed by 1-h of incubation in blocking solution (0.1 M phosphate-buffered saline (PBS) supplemented with 3% normal goat serum and 0.2% Triton X-100). Sections were then incubated overnight with Ki67 (1∶400 Novacastra), DCX (1∶150 Santa Cruz), and OX6 (major histocompatibility complex [MHC] class II; 1∶750 BD) antibody markers in PBS supplemented with 3% normal goat serum and 0.1% Triton X-100. Sections were subsequently washed and biotinylated secondary antibody (1∶200; Vector Laboratories, Burlingame, CA) in PBS supplemented with 3% normal goat serum, and 0.1% Triton X-100 was applied for 1 h. Next, the sections were incubated for 60 minutes in avidin–biotin substrate (ABC kit, Vector Laboratories, Burlingame, CA). All sections were then incubated for 1 minute in 3,30-diaminobenzidine (DAB) solution (Vector Laboratories). Sections were then mounted onto glass slides, dehydrated in ethanol and xylene, and cover-slipped using mounting medium.

### Stereological analysis

Immunohistochemistry techniques were used in order to tag three different cell markers. Activated microglia cells were visualized by staining with OX6, an antibody against antigen presenting cell; major histocompatibility complex class ll or MHC ll+. In order to determine the cell proliferation after chronic TBI, an antibody against Ki67 (protein present in all active cell cycle phases) was used [25]. Doublecortin (DCX), an antibody against immature migrating neurons, was used to determine neuronal proliferation. Moreover, positive stainings were analyzed with a Nikon Eclipse 600 microscope and quantified using Stereo Investigator software, version 10 (MicroBrightField, Colchester, VT). The estimated volume of OX6 positive cells was examined using Cavalieri estimator probe of the unbiased stereological cell technique [26] in analyzing the cortex, striatum, thalamus, cerebral peduncle, corpus callosum, and cerebellum areas such as white matter (WM), granular cell layer (GCL), and molecular layer (ML). Ki67 and DCX positive cells were counted within the subgranular zone (SGZ) in both hemispheres (ipsilateral and contralateral), using the optical fractionator probe of unbiased stereological cell counting technique. The sampling was optimized to count at least 300 cells per animal with error coefficients less than 0.07. Each counting frame (100 X 100 µm for OX6, Ki67, and DCX) was placed at an intersection of the lines forming a virtual grid (125 X 125 µm), which was randomly generated and placed by the software within the outlined structure.

### Statistical analysis

For data analyses, contralateral and ipsilateral corresponding brain areas were used as raw data providing 2 sets of data per treatment condition, therefore one-way analysis of variance (ANOVA) was used for group comparisons, followed by subsequent pairwise comparisons (post hoc tests Bonferonni test). All data are presented as mean values ± SEM. Statistical significance was set at *p*<0.05 for all analyses.

## Results

In the preliminary analyses of the data, comparisons between sham treatment ipsilateral and sham treatment contralateral side, across all brain regions studied, did not significantly differ (*p's*>0.05). Thus, the data from both sides of the sham treatment groups were combined. In addition, the contralateral side across all treatment groups also did not significantly differ (*p's*>0.05), thus analyses were focused on comparing the ipsilateral sides from treatment groups. Analyses revealed there were no significant differences between sham and PTSD alone treatment across all brain regions examined (*p's*>0.05).

### Upregulation of MHC ll+ activated microglia cells in chronic TBI alone and combined TBI-PTSD

**Figure 1:**
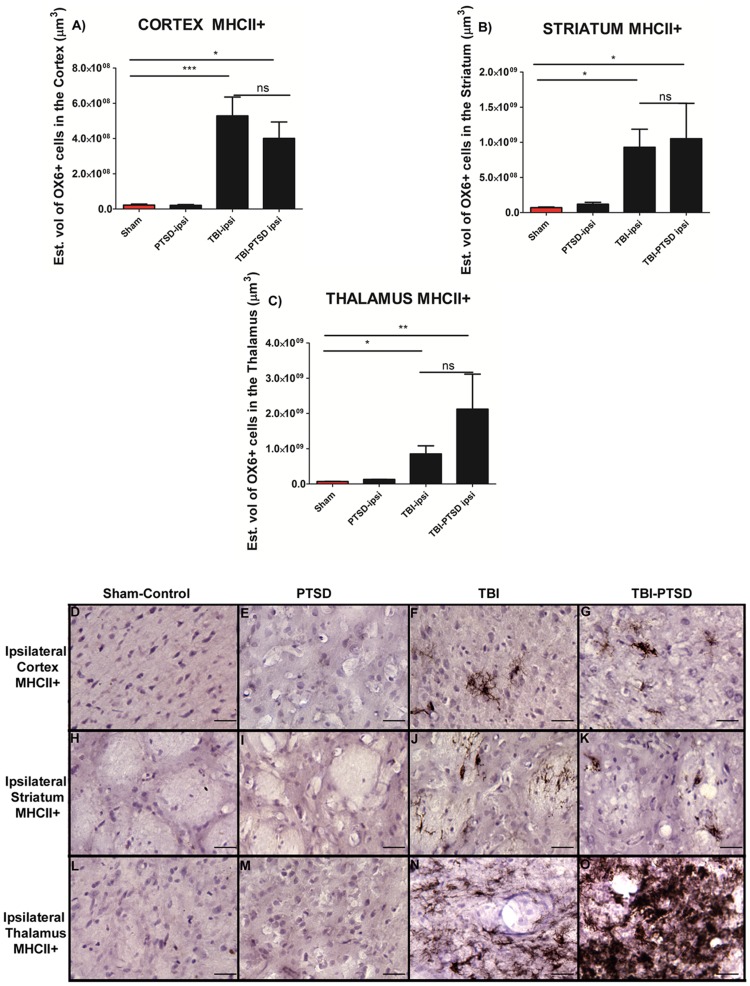
OX6 + expression in ipsilateral gray matter subcortical regions of TBI and TBI-PTSD rats. Figures 1A–C represent quantitative data of estimated volumes (µm^3^) of OX6+ in A) cortex, B) striatum, and C) thalamus. Figures 1D–G represent OX6+ immunostaining of the ipsilateral side of cortex in D) sham-no PTSD, E) sham-PTSD, F) TBI-PTSD, G) TBI. Figures H-K represent OX6+ immunostaining of the ipsilateral side of striatum in H) sham-no PTSD, I) sham-PTSD, J) TBI-PTSD, K) TBI. Figures L-O represent OX6+ immunostaining of the ipsilateral side of thalamus in L) sham-no PTSD, M) sham-PTSD, N) TBI-PTSD, O) TBI. Note that in Figure 1C, N, the upregulation of activated microglia cells reach significance only for the group of chronic TBI combined with PTSD. Cortex, F_3,20_ = 11.90,***p<0.0004; striatum, F_3,20_ = 6.629, **p<0.0036; thalamus, F_3,20_ = 5.999, ** *p*<0.0076. Scale bars for D, E, F, G, H, I, J, K, L, M, N, O are 1 µm.

To test the hypothesis of whether upregulation of microglia cells associated with chronic TBI was further exacerbated in a PTSD model with chronic TBI, different subcortical gray and WM areas were examined. The estimated volume of activated microglia cells (MHC ll+) was calculated using an anti-OX6 antibody. ANOVA revealed significant treatment effects in MHC II+ expression in the three brain regions examined (cortex, F_3,20_ = 11.90,***p<0.0004; striatum, F_3,20_ = 6.629, **p<0.0036; thalamus, F_3,20_ = 5.999, ** *p*<0.0076). Pairwise comparisons revealed TBI alone and combined TBI-PTSD resulted in a significant upregulation in the volume of MHC II-labeled activated microglia cells in gray matter areas ipsilateral to TBI when compared to PTSD alone and sham treatment (*p*<0.05) (Figure 1A, B, C). Moreover, TBI alone and combined TBI-PTSD, did not significantly differ between each other in the volume of MHC ll+ in ipsilateral cortex (Figure 1A), striatum (Figure 1B), and thalamus (Figure 1C) (*p<0.05*).

**Figure 2:**
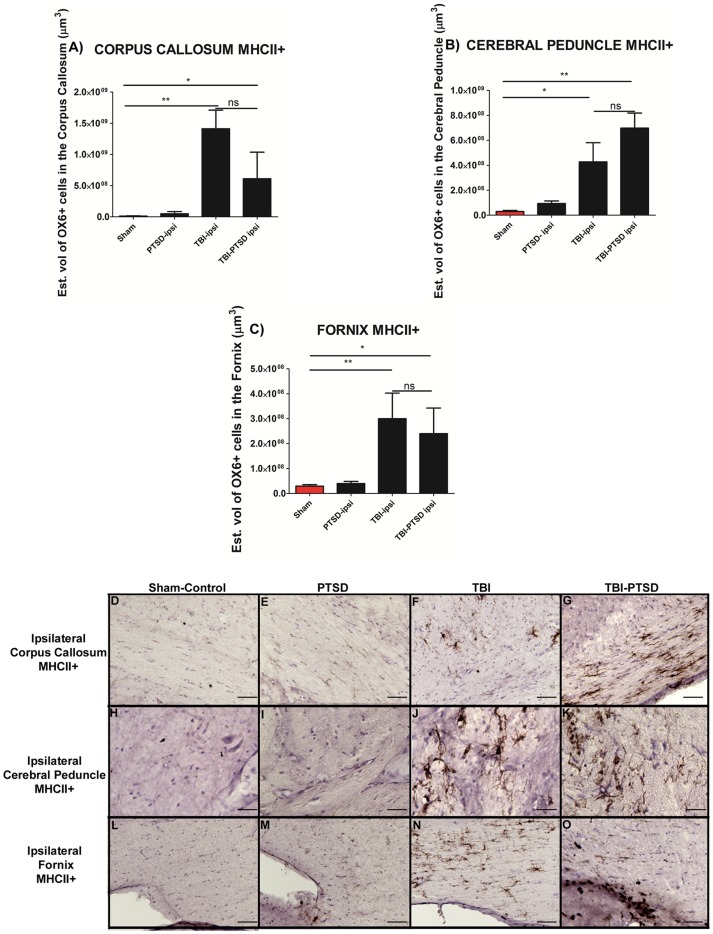
OX6 + expression in ipsilateral subcortical white matter regions of TBI and TBI-PTSD rats. Figures 2 A–C represent quantitative data of estimated volumes (µm^3^) of OX6+ in A) corpus callosum, B) cerebral peduncle, and C) fornix. Figures 2D–G represent OX6+ immunostaining of the ipsilateral side of corpus callosum in D) sham-no PTSD, E) sham-PTSD, F) TBI-PTSD, G) TBI. Figures 2 H–K represent OX6+ immunostaining of the ipsilateral side of cerebral peduncle in H) sham-no PTSD, I) sham-PTSD, J) TBI-PTSD, K) TBI. Figures2 L-O represent OX6+ immunostaining of the ipsilateral side of fornix in L) sham-no PTSD, M) sham-PTSD, N) TBI-PTSD, O) TBI. Corpus callosum, F_3,20_ = 8.611, **p<0.0017, cerebral peduncle F_3,20_ = 8.550, **p<0.002, fornix, F_3,20_ = 8.368, **p<0.002. Scale bars for D, E, F, G, H, I, J, K, L, M, N, O are 1 µm.

Further analysis showed significant treatment effects in MHC II+ expression in the white matter areas (corpus callosum, F_3,20_ = 8.611, **p<0.0017, cerebral peduncle F_3,20_ = 8.550, **p<0.002, fornix, F_3,20_ = 8.368, **p<0.002). Pairwise comparisons revealed that TBI alone and combined TBI-PTSD also instigated an increase of activated microglia cells (MHC ll+) volume in ipsilateral white matter areas compared with PTSD alone and sham treatment (*p's*<0.05) (Figure 2). Significant exacerbation of microglia cells in the cerebral peduncle was evident in the TBI and TBI-PTSD rats compared to PTSD alone and sham treatment (*p<0.05*). In addition, a significant increase in activated microglia cells in the fornix of TBI alone and TBI-PTSD group was found (p<0.05), relative to PTSD alone and sham treatment (p<0.05). TBI alone and TBI- PTSD resulted in an equivalent upregulation of activated microglia cells in corpus callosum, cerebral peduncle and fornix around the injury side (*p*<0.05) (Figure 2A, B, C).

**Figure 3:**
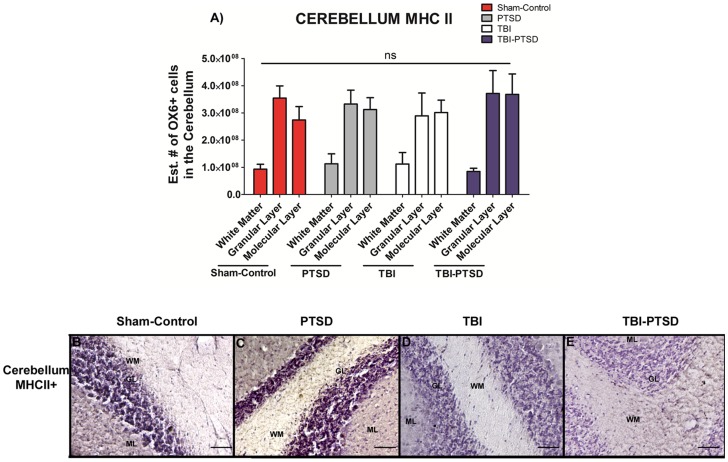
OX6 + expression in white matter, granular cell layer and molecular layer of the cerebellum of TBI and TBI-PTSD rats. Figure 3 A represents quantitative data of the estimated volumes (µm^3^) of OX6+ in three distinct regions of the cerebellum; white matter (WM), granular cell layer (GCL) and molecular layer (ML). Figures 3 B–F represent OX6+ immunostaining of the cerebellum in B) sham-no PTSD, C) sham-PTSD, D) TBI-PTSD, E) TBI. As shown in Figure 3 A, and in the photomicrographs B–E, there is no detectable upregulation of activated microglia cells in any of the examined cerebellar regions or across any of our treatment groups (sham-no PTSD, sham-PTSD, TBI-PTSD, and TBI alone); *p*>0.05. Scale bars for B-E are 1 µm.

The estimated volume of activated MHC II+ microglia cells was also calculated in the GCL, WM, and ML of the cerebellum. ANOVA revealed no detectable treatment effects on upregulation of activated microglia cells in any of the examined cerebellar regions known already the four treatment groups (Figure 3) (*p's*>0.05).

### Chronic TBI impairs hippocampal cell survival and proliferation, but not neuronal differentiation in neurogenic niches

**Figure 4:**
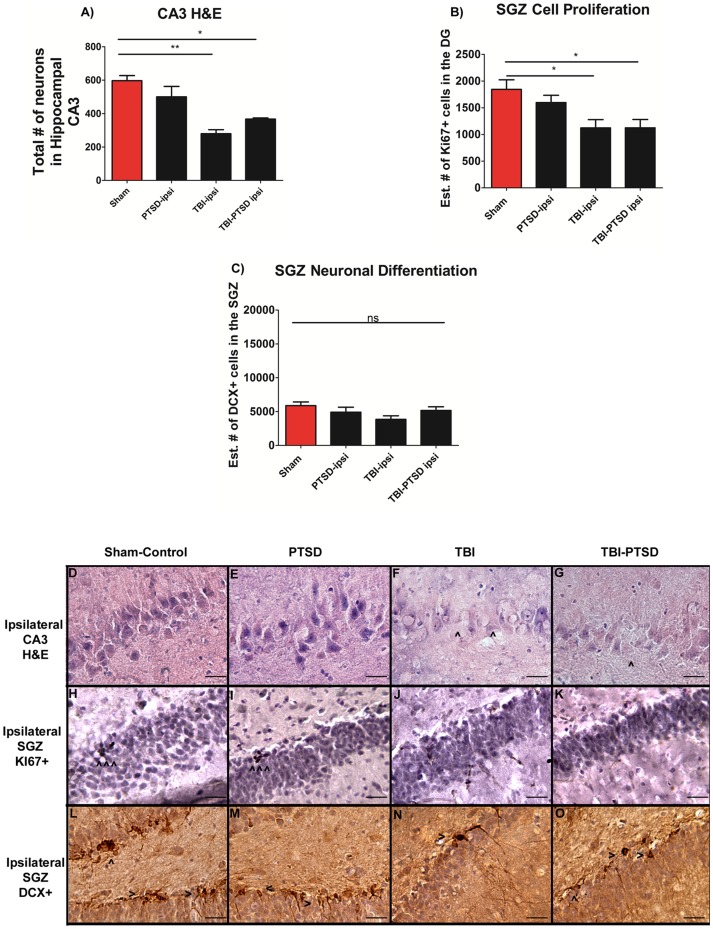
H&E, cell proliferation Ki67+, and neuronal differentiation DCX+ expressions in the hippocampus of TBI and TBI-PTSD rats. Figures 4 A–C represent quantitative data of A) total # of neurons in hippocampal CA3, B) estimated # of Ki67+ proliferating cells in the SGZ of the DG, and C) the estimated # of DCX+ migrating cells in the SGZ of the DG. Figures 4 D–G represent H&E staining of the ipsilateral hippocampal CA3 region in D) sham-no PTSD, E) sham-PTSD, F) TBI-PTSD, G) TBI. Figures 4 H-K represent Ki67+ immunostaining of the ipsilateral SGZ of the DG in H) sham-no PTSD, I) sham-PTSD, J) TBI-PTSD, K) TBI. Figures 4 L–O represent DCX+ immunostaining of the ipsilateral SGZ of the DG in L) sham-no PTSD, M) sham-PTSD, N) TBI-PTSD, O) TBI. CA3, F_3,8_ = 13.570, **p<0.0017, SGZ Ki67, F_3,20_ = 5.017, *p<0.0106, DCX, F_3,20_ = 1.959 *p*<0.1512. Scale bars for D–G are 50 µm and H–O are 1 µm.

**Figure 5:**
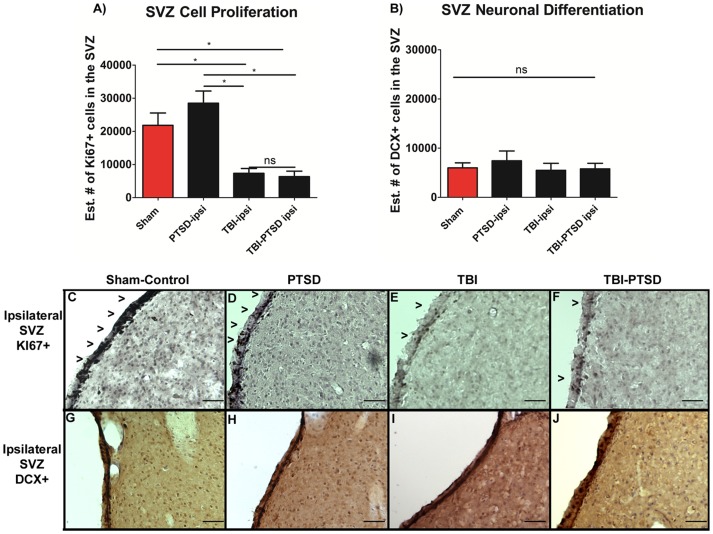
Cell proliferation Ki67+, and neuronal differentiation DCX+ expression in the SVZ of the lateral ventricle. Figures 5 A–B represent quantitative data of A) total # of Ki67+ cells representing the number of cell proliferating in the SVZ, B) the estimated # of DCX+ migrating cells in the SVZ of the lateral ventricle. Figures 5 C–F represent Ki67+ immunostaining of the ipsilateral SVZ in C) sham-no PTSD, D) sham-PTSD, E) TBI, F) TBI-PTSD. Figures 5 G–J represent DCX+ immunostaining of the ipsilateral SVZ of the lateral ventricle in G) sham-no PTSD, H) sham-PTSD, I) TBI, J) TBI-PTSD. SVZ Ki67, F_3,20_ = 7.863, **p<0.0012, SVZ DCX, F_3,20_ = 0.324, ns *p* = 0.8076. Scale bars for C–J are 50 µm.

Next, we examined the effects of PTSD on chronic TBI by evaluating the total number of surviving neurons in the hippocampal CA3 region, the estimated number of positive dividing cells within SGZ, and SVZ and the estimated number of positive neuronal differentiating cells within SGZ, and SVZ where examined. ANOVA revealed significant treatment effects on neuronal survival in hippocampal CA3 (F_3,8_ = 13.570, **p<0.0017), with post hoc tests demonstrating that both TBI alone and combined TBI-PTSD significantly reduced CA3 cell survival in the ipsilateral hippocampus relative to ipsilateral PTSD alone and sham treatment (*p's*<0.05) (Figure 4A). There was no significant difference in the number of surviving neurons in the CA3 between TBI alone and TBI-PTSD animals (*p>0.05*) (Figure 4A). Furthermore, analyses of cell proliferation, as evidenced by number of positive Ki67 cells in the SGZ of the hippocampus, and SVZ of the lateral ventricle revealed significant treatment effects (F_3,20_ = 5.017, *p<0.01, F_3,20_ = 7.863 **p<0.0012). Post hoc tests revealed that TBI alone and combined TBI –PTSD significantly reduced cell proliferation in SGZ and SVZ in a similar manner when compared to PTSD alone and sham treatment. Both TBI alone and combined TBI-PTSD prompted a decline of proliferating cells only in the ipsilateral side of SGZ and SVZ compared to the corresponding hemispheres of PTSD alone and sham treatment (Figure 4B, and Figure 5A) (*p*<0.05). Finally, ANOVA revealed no significant treatment effects (F_3,20_ = 1.9597 *ns p* = 0.1512, F_3,20_ = 0.324 *p* = 0.8076) on the neuronal differentiation in the SGZ and SVZ (Figure 4C, Figure 5B). Interestingly, while cell survival and proliferation were altered by TBI and combined TBI-PTSD, there were no significant differences produced by these injuries on the neuronal differentiation across all treatment groups (*p*>0.05).

## Discussion

In the present in vivo study, we demonstrated that exacerbation of activated microglia cells was detected at 8 weeks in chronic TBI and is associated with CA3 cell loss, and dysfunctional cell proliferation in the hippocampus. In this first assessment of histological pathology, we have found that PTSD did not exacerbate TBI-induced neuroinflammation and neurodegeneration. After eight weeks post-TBI, chronic TBI alone, and chronic TBI combined with PTSD showed a similar significant augmentation of intensified activated microglia cells in cortical and subcortical regions.

There was a 20-fold increase of activated microglia cells in cortex for TBI alone and TBI combined with PTSD when compared to PTSD alone and sham treatment. There was a 12-fold increase of activated microglia cells in both TBI and TBI-PTSD in the striatum. The TBI-PTSD group showed 30-fold increase relative to sham treatment; however, it failed to reach statistical significance when compared with TBI alone group. For the TBI alone and TBI-PTSD, there were 10-fold and 30-fold increments, respectively, of activated microglia cells present in the thalamus. The corpus callosum showed 100-fold increases of active microglia cells compared with PTSD alone and sham treatment. In the cerebral peduncle area, there were 10-fold and 15-fold increments in TBI and TBI-PTSD, respectively, relative to PTSD alone and sham treatment. The area of the hippocampal fornix showed 10-fold and 8-fold increments in TBI and TBI-PTSD, respectively, compared with PTSD alone and sham treatment. Furthermore, cerebellar analysis showed that there were no significant increments of activated microglia cells in the white matter (WM), granular cell layer (GCL), and molecular layer (ML) relative to sham treatment. In parallel, distinct hippocampal areas were assessed to determine neurodegeneration. Both TBI and TBI-PTSD showed significant declines of CA3 hippocampal neurons relative to stress and sham treatment. By comparing our TBI model with the TBI-PTSD model, we can conclude that chronic PTSD exposure did not further generate an increase in neuronal cell death in the CA3 region of the hippocampus. Of note, analysis of the contralateral side showed no significant differences in CA3 cell loss across treatment groups.

Further examination of the hippocampus revealed significant declines in the proliferative capacity of newly born cells within the SGZ. There was about 40% decrease in cell proliferation within the SGZ in both TBI and TBI-PTSD when compared with PTSD alone and sham treatment. The analysis showed that the injury caused by TBI alone was associated with impaired cell differentiation in the SGZ of the hippocampus since there was no further impairment on cell proliferation when TBI is combined with PTSD. Moreover, histological analysis of the SVZ showed that the proliferative ability of the cells in this neurogenic niche was also affected by chronic TBI alone which was not further impaired when TBI was combined with PTSD. Of note, in both neurogenic niches, SGZ and SVZ, the cell differentiation profile was not affected by either TBI alone or by the combination of TBI and PTSD.

To our knowledge, experiments using animal models of TBI exposed to PTSD to evaluate synergistic effects in neuroinflammation and neuronal degeneration are currently lacking, representing a major gap of knowledge on the interaction between TBI and psychological morbidities, such as PTSD. There is one study that assessed the influence of chronic stress on blast-related traumatic brain injury in rats [27]. As in the current work, these investigators documented brain injury in response to stress and TBI. However, as they did not include a non-stress TBI group, it cannot be confirmed that stress exacerbated the histopathology they observed. Thus, the issue of the conditions, brain areas and aspects of histopathology affected by stress-TBI interactions remains unresolved. This lack of conclusive scientific evidence for stress-TBI interactions also highlights the complexity behind the pathological mechanisms that link chronic head traumas and PTSD in TBI survivors. Nonetheless, while the present observations do not show PTSD exacerbation of TBI-induced histopathological deficits, these findings provide compelling results that can be used to guide future studies on the biological bases of TBI and its association with the development of neuropsychological morbidities post-TBI or the worsening of pre-existing conditions. For example, the current work focused on assessment of histological pathology in response to PTSD and brain trauma. It will be important in subsequent work to include an analysis of how stress-TBI interactions are expressed at behavioral and cognitive levels, in conjunction with histopathological assessments.

Long-term consequences of chronic TBI may be related to increased risk for chronic neuroinflammation, neurodegenerative diseases, executive function impairments, and the development of neuropsychological disorders such as anxiety, dementia, depression and PTSD [5], [6], [13], [23], [28], [29], [30], [31], [32], [33]. A growing body of literature suggests that TBI and PTSD are becoming the signature morbidities of our military personnel and veterans [5], [6], [25]. Clinical studies show that TBI is significantly associated with limited functional impairments, while TBI comorbid with PTSD and depression was significantly associated with chronic long lasting cognitive deficits in servicemen following deployment [5], [6]. In addition, animal studies show that exposure to blast injuries induced psychological abnormalities and increments of proteins that enhance fear responses for several months after the initial exposure [32]. In the clinic, patients with a history of brain injury display neuropsychological disturbances affecting executive function, attention and memory [34], which may be mediated by reduced cerebral blood flow in the thalamus, a brain structure implicated in neurological impairments such as memory and learning and verbal respond speed [35]. These findings are in agreement with the present results which depict a significant exacerbation of activated microglia cells in dorsal thalamic nuclei in animals exposed to TBI combined with PTSD.

The present results also showed that TBI and TBI with PTSD produced an equal extent of hippocampal neurodegeneration. However, PTSD alone did not alter the hippocampal CA3 survival. Similarly, TBI and combined TBI- PTSD, but not PTSD alone, reduced the cellular proliferative capacity in both neurogenic niches of SGZ and SVZ, while neuronal differentiation was not significantly impaired in any of the treatment groups. These results support previous clinical studies, showing a lack of decrease in hippocampal volume in TBI-PTSD patients [15], [36], [37], [38], [39], [40], [41], [42], [43]. Our findings are in agreement with these clinical studies, whereby chronic stress did not exacerbate the neuropathological effects of TBI alone. However, these findings warrant further investigations in view of previous work demonstrating that stress, particularly elevated levels of corticosterone, can exacerbate damage to the hippocampus in response to metabolic insults, including hypoxia or administration of neurotoxins [44]. Thus, whether the reported stress-induced exacerbation of brain damage from metabolic challenges can be distinguished from potential stress-TBI interactions remains to be determined.

Finally, clinical studies have found that there is a close association between patients that suffer TBI-and a traumatic psychological event, with decreased hippocampal neuronal volume. In addition, these studies showed that these patients have an increased risk for the development of more severe neuropsychological disorders such as severe depression, bipolar disorder and PTSD [15]. There is also evidence suggesting that there is a strong link between TBI and worsening of psychological conditions such as depression and PTSD[15], [45], [46], [47], [48], [49], [50], [51], [52], [53]. To this end, whether hippocampal cell loss and alteration in neurogenic capacity are associated with the development of TBI co-morbidities is not clear. Further studies addressing the biological bases on how TBI combined with neuropsychological stress and more severe PTSD impairs hippocampal function, such as long-term potentiation and memory formation, are needed.

## Conclusions

To our knowledge, this is the first laboratory report of histopathological characterization of TBI-PTSD. This study reveals that the combined TBI-PTSD group displayed similar neuroinflammation and impaired cell proliferation profiles. Other TBI-mediated cell death events, such as oxidative stress and apoptosis, warrant further investigations. Investigations of TBI and its co-morbidity factors will allow a better understanding of the disease pathology and guide treatment that will address both primary and secondary cell death events, as well as psychological and physical impairments.

## References

[1] Faul M, Xu L, Wald MM, Coronado VG (2010) Traumatic brain injury in the United States: Emergency department visits, hospitalizations and deaths. Atlanta (GA): Centers for Disease Control and Prevention, National Center for Injury Prevention and Control.

[2] Sosin DM, Sniezek JE, Waxweiler RJ (1995) Trends in death associated with traumatic brain injury, 1979 through 1992. Success and failure. JAMA 273: 1778–1780. 7769773

[3] Coronado VG, Xu L, Basavaraju SV, McGuire LC, Wald MM, et al (2011) Surveillance for traumatic brain injury-related deaths–United States, 1997–2007. MMWR Surveill Summ 60: 1–32. 21544045

[4] Kraus JF (1993) Epidemiology of head injury. In: Cooper PR, editor. Head Injury, 3rd ed. Baltimore: Williams and Wilkins. pp. 1–25.

[5] Rogers JM, Read CA (2007) Psychiatric comorbidity following traumatic brain injury. Brain Inj 21: 1321–1333. 18066935 10.1080/02699050701765700

[6] Vasterling JJ, Brailey K, Proctor SP, Kane R, Heeren T, et al (2012) Neuropsychological outcomes of mild traumatic brain injury, post-traumatic stress disorder and depression in Iraq-deployed US Army soldiers. Br J Psychiatry 201: 186–192. 22743844 10.1192/bjp.bp.111.096461

[7] Rona RJ, Jones M, Fear NT, Hull L, Murphy D, et al (2012) Mild traumatic brain injury in UK military personnel returning from Afghanistan and Iraq: cohort and cross-sectional analyses. J Head Trauma Rehabil 27: 33–44. 22241066 10.1097/HTR.0b013e318212f814

[8] Hoge CW, McGurk D, Thomas JL, Cox AL, Engel CC, et al (2008) Mild traumatic brain injury in U.S. Soldiers returning from Iraq. N Engl J Med 358: 453–463. 18234750 10.1056/NEJMoa072972

[9] Tanielian T, Jaycox LH (2008) Wounds of war: Psychological and cognitive injuries, their consequences, and services to assist recovery. RAND Corporation. MG-720-CCF. Available: http://www.rand.org/pubs/monographs/MG720. Accessed 2013 Nov 5.

[10] Wagner AK, Kline AE, Ren D, Willard LA, Wenger MK, et al (2007) Gender associations with chronic methylphenidate treatment and behavioral performance following experimental traumatic brain injury. Behav Brain Res 181: 200–209. 17517440 10.1016/j.bbr.2007.04.006 PMC1974874

[11] Holschneider DP, Guo Y, Roch M, Norman KM, Scremin OU (2011) Acetylcholinesterase inhibition and locomotor function after motor-sensory cortex impact injury. J Neurotrauma 28: 1909–1919. 21787180 10.1089/neu.2011.1978

[12] Potts MB, Adwanikar H, Noble-Haeusslein LJ (2009) Models of traumatic cerebellar injury. Cerebellum 8: 211–221. 19495901 10.1007/s12311-009-0114-8 PMC2734258

[13] Gao X, Deng-Bryant Y, Cho W, Carrico KM, Hall ED, et al (2008) Selective death of newborn neurons in hippocampal dentate gyrus following moderate experimental traumatic brain injury. J Neurosci Res 86: 2258–2270. 18381764 10.1002/jnr.21677 PMC3757515

[14] Rola R, Mizumatsu S, Otsuka S, Morhardt DR, Noble-Haeusslein LJ, et al (2006) Alterations in hippocampal neurogenesis following traumatic brain injury in mice. Exp Neurol 202: 189–199. 16876159 10.1016/j.expneurol.2006.05.034

[15] Shin LM, Rauch SL, Pitman RK (2006) Amygdala, medial prefrontal cortex, and hippocampal function in PTSD. Ann N Y Acad Sci 1071: 67–79. 16891563 10.1196/annals.1364.007

[16] Zoladz PR, Fleshner M, Diamond DM (2012) Psychosocial animal model of PTSD produces a long-lasting traumatic memory, an increase in general anxiety and PTSD-like glucocorticoid abnormalities. Psychoneuroendocrinology 37: 1531–1545. 22421563 10.1016/j.psyneuen.2012.02.007

[17] Roth TL, Zoladz PR, Sweatt JD, Diamond DM (2011) Epigenetic modification of hippocampal Bdnf DNA in adult rats in an animal model of post-traumatic stress disorder. J Psychiatr Res 45: 919–926. 21306736 10.1016/j.jpsychires.2011.01.013 PMC3335738

[18] Zoladz PR, Conrad CD, Fleshner M, Diamond DM (2008) Acute episodes of predator exposure in conjunction with chronic social instability as an animal model of post-traumatic stress disorder. Stress 11: 259–281. 18574787 10.1080/10253890701768613 PMC2535807

[19] Park CR, Campbell AM, Diamond DM (2001) Chronic psychosocial stress impairs learning and memory and increases sensitivity to yohimbine in adult rats. Biol Psychiatry 50: 994–1004. 11750896 10.1016/s0006-3223(01)01255-0

[20] Diamond DM, Park CR, Heman KL, Rose GM (1999) Exposing rats to a predator impairs spatial working memory in the radial arm water maze. Hippocampus 9: 542–552. 10560925 10.1002/(SICI)1098-1063(1999)9:5<542::AID-HIPO8>3.0.CO;2-N

[21] Diamond DM, Zoladz PR (2010) An animal model of PTSD which integrates inescapable predator exposure and social instability. Culture Psy Neurosciences 15: 6–7.

[22] Glover LE, Tajiri N, Lau T, Kaneko Y, van Loveren H, et al (2012) Immediate, but not delayed, microsurgical skull reconstruction exacerbates brain damage in experimental traumatic brain injury model. PLoS One 7: e33646. 22438975 10.1371/journal.pone.0033646 PMC3306278

[23] Yu S, Kaneko Y, Bae E, Stahl CE, Wang Y, et al (2009) Severity of controlled cortical impact traumatic brain injury in rats and mice dictates degree of behavioral deficits. Brain Res 1287: 157–163. 19573519 10.1016/j.brainres.2009.06.067

[24] Hayashi T, Kaneko Y, Yu S, Bae E, Stahl CE, et al (2009) Quantitative analyses of matrix metalloproteinase activity after traumatic brain injury in adult rats. Brain Res 1280: 172–177. 19464272 10.1016/j.brainres.2009.05.040

[25] Scholzen T, Gerdes J (2000) The Ki-67 protein: from the known and the unknown. J Cell Physiol 182: 311–322. 10653597 10.1002/(SICI)1097-4652(200003)182:3<311::AID-JCP1>3.0.CO;2-9

[26] Mayhew TM (1991) The new stereological methods for interpreting functional morphology from slices of cells and organs. Exp Physiol 76: 639–665. 1742008 10.1113/expphysiol.1991.sp003533

[27] Kwon SK, Kovesdi E, Gyorgy AB, Wingo D, Kamnaksh A, et al (2011) Stress and traumatic brain injury: a behavioral, proteomics, and histological study. Front Neurol 2: 12. 21441982 10.3389/fneur.2011.00012 PMC3057553

[28] Gao X, Deng P, Xu ZC, Chen J (2011) Moderate traumatic brain injury causes acute dendritic and synaptic degeneration in the hippocampal dentate gyrus. PLoS One 6: e24566. 21931758 10.1371/journal.pone.0024566 PMC3172233

[29] Yang J, You Z, Kim HH, Hwang SK, Khuman J, et al (2010) Genetic analysis of the role of tumor necrosis factor receptors in functional outcome after traumatic brain injury in mice. J Neurotrauma 27: 1037–1046. 20205514 10.1089/neu.2009.1229 PMC2943499

[30] Harting MT, Jimenez F, Adams SD, Mercer DW, Cox CS Jr (2008) Acute, regional inflammatory response after traumatic brain injury: Implications for cellular therapy. Surgery 144: 803–813. 19081024 10.1016/j.surg.2008.05.017 PMC3774544

[31] Kamnaksh A, Kovesdi E, Kwon SK, Wingo D, Ahmed F, et al (2011) Factors affecting blast traumatic brain injury. J Neurotrauma 28: 2145–2153. 21861635 10.1089/neu.2011.1983

[32] Elder GA, Dorr NP, De Gasperi R, Gama Sosa MA, Shaughness MC, et al (2012) Blast exposure induces post-traumatic stress disorder-related traits in a rat model of mild traumatic brain injury. J Neurotrauma 29: 2564–2575. 22780833 10.1089/neu.2012.2510 PMC3495123

[33] Glaesser J, Neuner F, Lutgehetmann R, Schmidt R, Elbert T (2004) Posttraumatic Stress Disorder in patients with traumatic brain injury. BMC Psychiatry 4: 5. 15113439 10.1186/1471-244X-4-5 PMC395832

[34] Little DM, Kraus MF, Joseph J, Geary EK, Susmaras T, et al (2010) Thalamic integrity underlies executive dysfunction in traumatic brain injury. Neurology 74: 558–564. 20089945 10.1212/WNL.0b013e3181cff5d5 PMC2830915

[35] Strangman GE, Goldstein R, O'Neil-Pirozzi TM, Kelkar K, Supelana C, et al (2009) Neurophysiological alterations during strategy-based verbal learning in traumatic brain injury. Neurorehabil Neural Repair 23: 226–236. 19047359 10.1177/1545968308324225

[36] Carrion VG, Weems CF, Eliez S, Patwardhan A, Brown W, et al (2001) Attenuation of frontal asymmetry in pediatric posttraumatic stress disorder. Biol Psychiatry 50: 943–951. 11750890 10.1016/s0006-3223(01)01218-5

[37] De Bellis MD, Keshavan MS, Shifflett H, Iyengar S, Beers SR, et al (2002) Brain structures in pediatric maltreatment-related posttraumatic stress disorder: a sociodemographically matched study. Biol Psychiatry 52: 1066–1078.38. 12460690 10.1016/s0006-3223(02)01459-2

[38] Fennema-Notestine C, Stein MB, Kennedy CM, Archibald SL, Jernigan TL (2002) Brain morphometry in female victims of intimate partner violence with and without posttraumatic stress disorder. Biol Psychiatry 52: 1089–1101. 12460692 10.1016/s0006-3223(02)01413-0

[39] De Bellis MD, Keshavan MS, Clark DB, Casey BJ, Giedd JN, et al (1999) A.E. Bennett Research Award. Developmental traumatology. Part II: Brain development. Biol Psychiatry 45: 1271–1284. 10349033 10.1016/s0006-3223(99)00045-1

[40] Golier JA, Yehuda R, De Santi S, Segal S, Dolan S, et al (2005) Absence of hippocampal volume differences in survivors of the Nazi Holocaust with and without posttraumatic stress disorder. Psychiatry Res 139: 53–64. 15939577 10.1016/j.pscychresns.2005.02.007

[41] Pederson CL, Maurer SH, Kaminski PL, Zander KA, Peters CM, et al (2004) Hippocampal volume and memory performance in a community-based sample of women with posttraumatic stress disorder secondary to child abuse. J Trauma Stress 17: 37–40. 15027791 10.1023/B:JOTS.0000014674.84517.46

[42] Bonne O, Brandes D, Gilboa A, Gomori JM, Shenton ME, et al (2001) Longitudinal MRI study of hippocampal volume in trauma survivors with PTSD. Am J Psychiatry 158: 1248–1251. 11481158 10.1176/appi.ajp.158.8.1248 PMC2819102

[43] De Bellis MD, Hall J, Boring AM, Frustaci K, Moritz G (2001) A pilot longitudinal study of hippocampal volumes in pediatric maltreatment-related posttraumatic stress disorder. Biol Psychiatry 50: 305–309. 11522266 10.1016/s0006-3223(01)01105-2

[44] Sapolsky RM (1999) Glucocorticoids, stress, and their adverse neurological effects: relevance to aging. Exp Gerontol 34: 721–732. 10579633 10.1016/s0531-5565(99)00047-9

[45] Pardini M, Krueger F, Koenigs M, Raymont V, Hodgkinson C, et al (2012) Fatty-acid amide hydrolase polymorphisms and post-traumatic stress disorder after penetrating brain injury. Transl Psychiatry 2: e75. 22832737 10.1038/tp.2012.1 PMC3309545

[46] Bremner JD, Vythilingam M, Vermetten E, Southwick SM, McGlashan T, et al (2003) MRI and PET study of deficits in hippocampal structure and function in women with childhood sexual abuse and posttraumatic stress disorder. Am J Psychiatry 160: 924–932. 12727697 10.1176/appi.ajp.160.5.924

[47] Gurvits TV, Shenton ME, Hokama H, Ohta H, Lasko NB, et al (1996) Magnetic resonance imaging study of hippocampal volume in chronic, combat-related posttraumatic stress disorder. Biol Psychiatry 40: 1091–1099. 8931911 10.1016/S0006-3223(96)00229-6 PMC2910907

[48] Gilbertson MW, Shenton ME, Ciszewski A, Kasai K, Lasko NB, et al (2002) Smaller hippocampal volume predicts pathologic vulnerability to psychological trauma. Nat Neurosci 5: 1242–1247. 12379862 10.1038/nn958 PMC2819093

[49] Brown S, Freeman T, Kimbrell T, Cardwell D, Komoroski R (2003) In vivo proton magnetic resonance spectroscopy of the medial temporal lobes of former prisoners of war with and without posttraumatic stress disorder. J Neuropsychiatry Clin Neurosci 15: 367–370. 12928515 10.1176/jnp.15.3.367

[50] Freeman TW, Cardwell D, Karson CN, Komoroski RA (1998) In vivo proton magnetic resonance spectroscopy of the medial temporal lobes of subjects with combat-related posttraumatic stress disorder. Magn Reson Med 40: 66–71. 9660555 10.1002/mrm.1910400110

[51] Mohanakrishnan Menon P, Nasrallah HA, Lyons JA, Scott MF, Liberto V (2003) Single-voxel proton MR spectroscopy of right versus left hippocampi in PTSD. Psychiatry Res 123: 101–108. 12850249 10.1016/s0925-4927(03)00044-1

[52] Schuff N, Neylan TC, Lenoci MA, Du AT, Weiss DS, et al (2001) Decreased hippocampal N-acetylaspartate in the absence of atrophy in posttraumatic stress disorder. Biol Psychiatry 50: 952–959. 11750891 10.1016/s0006-3223(01)01245-8 PMC2733624

[53] Villarreal G, Petropoulos H, Hamilton DA, Rowland LM, Horan WP, et al (2002) Proton magnetic resonance spectroscopy of the hippocampus and occipital white matter in PTSD: preliminary results. Can J Psychiatry 47: 666–670. 12355679 10.1177/070674370204700709

